# Alexithymia and Alcohol Use: Evaluating the Role of Interoceptive Sensibility with the Revised Multidimensional Assessment of Interoceptive Awareness

**DOI:** 10.1007/s10862-023-10034-y

**Published:** 2023-04-04

**Authors:** Michael Lyvers, Fred Arne Thorberg

**Affiliations:** Bond University, 4229 Gold Coast, QLD Australia

**Keywords:** Alcohol, Alexithymia, Emotion regulation, Interoception

## Abstract

Alexithymia has been linked to risky or problematic alcohol use, with a common interpretation invoking deficient emotion regulation and use of alcohol to cope with distress. An alternative explanation positing a general deficit of interoception in alexithymia suggested that poor awareness of internal cues of overconsumption may promote excessive drinking. The present study assessed predictions based on these hypotheses in 337 young adult alcohol users recruited online. Participants completed validated questionnaire indices of alcohol use, alexithymia, emotion regulation, interoceptive sensibility, and sensitivity to reward and punishment. Alcohol use was positively correlated with alexithymia and reward sensitivity, and negatively correlated with emotion regulation as expected, but was uncorrelated with interoceptive sensibility. Alexithymia was not significantly correlated with most dimensions of interoceptive sensibility but was highly negatively correlated with emotion regulation. Hierarchical regression controlling for demographic variables indicated that alexithymia, emotion regulation, sex, and sensitivity to reward and punishment were significant predictors of alcohol use levels. Bootstrapped mediation test controlling for all other variables indicated mediation of the association between alexithymia and alcohol use by deficient emotion regulation but not interoceptive sensibility. Results supported the emotion regulation deficit interpretation of the association of alexithymia with alcohol use. Limitations concerning interoception measurement, online samples, self-report measures, cross-sectional designs, and collection of data during the COVID-19 pandemic are discussed. Future research could follow up on these findings by testing interoceptive accuracy in addition to interoceptive sensibility in relation to alexithymia and alcohol use.

## Introduction

Alexithymia is a trait dimension defined by difficulties identifying and describing feelings and an externally oriented thinking style (Bagby et al., 2020). Alexithymia has been linked to risky or problematic alcohol use in both clinical alcohol use disorder (AUD) samples (Cruise & Becerra, 2018; Thorberg et al., 2009) and in nonclinical samples of young adult social drinkers (Lyvers et al., 2018a, b, 2019, 2020). Alexithymia has also been linked to other addictive behaviors including risky or problematic use of other drugs (Ghalehban & Besharat, 2011; Lyvers et al., 2013, 2014), binge eating (Marsero et al., 2011; Westwood et al., 2017; Wheeler et al., 2005), pathological gambling (Marchetti et al., 2019; Toneatto et al., 2009), compulsive buying (Rose & Segrist, 2012), internet addiction (Kandri et al., 2014; Lyvers et al., 2016; Mahapatra & Sharma, 2018), and exercise addiction (Lyvers et al., 2021; Van Landeghem et al., 2019).

A common interpretation of the association of alexithymia with addictive behaviors in such research is that alexithymic people have difficulty self-regulating their emotions, and thus often turn to maladaptive coping strategies to alleviate distress. In other words, people who have difficulty identifying and describing their emotions would likely find it difficult to cope with negative moods via self-regulatory strategies such as problem-focused coping, reappraisal, attention shifting, or activating positive emotions (Wiśniewskiet al., 2021), and may learn to rely on substance use or other externalized behaviors to cope with distress. For example, Lyvers et al. (2018a) reported that alexithymia was linked to risky drinking through the specific drinking motive of drinking to cope with negative moods, presumably reflecting a deficit of emotional self-regulation in alexithymia and associated use of externalized means to cope with distress. By contrast, another trait that has been consistently linked to addictive behaviors - reward sensitivity (Dawe & Loxton, 2004) - was independently linked to risky drinking through the drinking motive of positive mood enhancement, presumably reflecting inherent responsivity of subcortical dopaminergic systems underlying reward drive.

An interesting alternative possibility to the emotion regulation deficit interpretation of alexithymia’s link to alcohol misuse was offered by Brewer et al. (2016), who proposed that alexithymia reflects a fundamental deficit of interoception that encompasses poor awareness not only of internal bodily manifestations of emotional states, but also non-affective internal cues of overconsumption, perhaps accounting for the associations of alexithymia with risky or problematic alcohol use. Interoception refers to sensing, processing and appraisal of internal bodily signals (Craig, 2009). Recent reviews (Khalsa et al., 2017; Lovelock et al., 2021; Suksasilp & Garfinkel, 2022) have highlighted the complex, multifaceted nature of interoception, the various neural systems and levels of processing involved, and the importance of a multidimensional approach to measurement. Regarding the latter, Garfinkel et al. (2015) distinguished between interoceptive accuracy, typically measured by a heartbeat counting task; interoceptive sensibility, the self-reported typical awareness of internal bodily sensations (the type of measurement used in the present study); and interoceptive metacognitive awareness, the degree of match or mismatch between confidence in interoceptive judgements and objective interoceptive accuracy. A subsequent theoretical review by Suksasilp and Garfinkel (2022) identified additional dimensions or levels of interoception to these, encompassing brain representation of bodily signals, their preconscious impacts, the degree of conscious attention directed to such signals, and their conscious interpretation. A further complexity noted by these authors was the likelihood of variation across different physiological systems of the body (e.g., cardiac vs. gustatory) within the same individual.

Given such complexity, it is perhaps not surprising that there is mixed research evidence suggesting interoceptive deficits associated with alexithymia or alcohol misuse. For example, alexithymia has been reported to be associated with poorer interoceptive accuracy in perception of heartbeats (Herbert et al., 2011; Murphy et al., 2018), though the validity of the heartbeat counting task as an index of interoceptive accuracy has recently been questioned (Desmedt et al., 2018; Ring & Brener, 2018; Ring et al., 2015; Zamariola et al., 2018). By contrast, Betka et al. (2017) found that alexithymia was positively correlated with both self-reported alcohol consumption and sensitivity to body sensations as assessed by the Body Perception Questionnaire (Porges, 1993), an index of interoceptive sensibility. Reflecting the inconsistency of the literature to date, a recent meta-analysis by Trevisan et al. (2019), including 66 published studies and encompassing measures of interoceptive accuracy and sensibility, found only a weak overall negative relationship between alexithymia and interoception. There was no overall association of alexithymia with interoceptive accuracy; however, as the authors noted, 28 of the 32 included studies had used the problematic heartbeat perception task. The efforts by Murphy et al. (2018) to statistically control for 10 potentially confounding variables still found only a weak negative relationship of alexithymia with heartbeat counting accuracy. Trevisan et al.’s meta-analysis found only marginally better support for a negative relationship of interoceptive sensibility with alexithymia as measured by the widely used Toronto Alexithymia Scale (TAS-20; Bagby et al., 1994a, b), with four studies reporting such a relationship using the Multidimensional Assessment of Interoceptive Awareness (MAIA; Mehling et al., 2012); on the other hand, six studies using a different self-report measure, the Body Perception Questionnaire (BPQ; Porges, 1993), found the opposite relationship. Trevisan et al. noted that there was stronger overall evidence for interoceptive deficits associated with alexithymia in binge eating disorder and bulimia, which are perhaps more relevant to the Brewer et al. (2016) hypothesis considered here in regard to alcohol use, such that poor sensitivity to internal cues of overconsumption was invoked as a contributing factor to excessive drinking.

Regarding the hypothesized link between deficient interoception and alcohol misuse, Col et al. (2016) reported that abstinent AUD patients showed poorer accuracy on the heartbeat counting task compared to controls. Jakubczyk et al. (2019) similarly reported that compared to healthy controls, AUD patients showed poorer heartbeat counting accuracy; however, they scored higher on a questionnaire measure of interoceptive sensibility (Private Body Consciousness scale), showing a dissociation between the two types of measures. On the other hand, results of an experiment by Leganes-Fonteneau et al. (2019) indicated that better discrimination of timing of heartbeats from that of tones was associated with higher levels of self-reported light-headedness induced by an acute alcohol dose, suggesting convergence between interoceptive heartbeat perception and interoceptive awareness of internal cues of intoxication as assessed by self-report. Interestingly, weak self-reported subjective response to negative effects of alcohol intoxication has long been implicated as a risk factor for problematic drinking (Schuckit et al., 2009). The relevance of this for alexithymia’s relationship with alcohol misuse is unclear, however; for example, a study of alcohol expectancies in AUD patients found that alexithymia was associated with stronger beliefs in negative effects of alcohol intoxication, in addition to increased alcohol-induced assertiveness in social situations (Thorberg et al., 2016). A recent review by Wiśniewskiet al. (2021) concluded that based on limited findings to date, deficits in interoceptive accuracy, but elevated interoceptive sensibility, may underlie some features of AUD such as alcohol craving and impaired emotion regulation; this could reflect bidirectional influences between risk factors and the neurotoxic effects of heavy alcohol consumption on brain regions involved in both interoception and emotion, such as the insula (also see review by Lovelock et al., 2021). Some of the same brain areas have been reported to show abnormalities in alexithymia, including the insula and frontal regions implicated in both interoception and emotions (Gu et al., 2013; Stevens et al., 2011; Xu et al., 2018), processes that have been theoretically linked to each other since the 19th century (Dewey, 1894). However, given the complexities of interoception and its measurement, evidence to date appears to be somewhat equivocal concerning the hypothesis that a general interoceptive deficit - whether measured objectively by accuracy or subjectively by self-report - is present in, and contributes to, alexithymia and its association with risky or problematic drinking.

The present study administered the revised MAIA (MAIA-2; Mehling et al., 2018) as the most comprehensive self-report index of interoceptive sensibility available to date, in addition to the widely used TAS-20 self-report index of alexithymia. The primary goal was to see if deficient interoceptive sensibility might show evidence of a mediating role in the link between alexithymia as measured by TAS-20 and alcohol use as measured by the Alcohol Use Disorders Identification Test (AUDIT; Babor et al., 2001), a widely used screening tool for alcohol problems. This approach was also deemed justified given the hypothesized role of deficient interoceptive sensibility in disordered behaviors more generally (Trevisan et al., 2019). Specifically, the primary goal of the present study was to assess whether a general deficit in interoceptive sensibility as indexed by MAIA-2 might be implicated in the link between alexithymia and alcohol use beyond interoception’s purported role in emotion regulation. This would be in line with the hypothesis of Brewer et al. (2016) that alexithymia reflects a general deficit of interoception that encompasses non-affective interoception such as sensitivity to satiety or intoxication cues. The present study attempted to test predictions of this hypothesis in alcohol-using young Australian adults, a group with high levels of risky alcohol use among alcohol users (Australian Institute of Health and Welfare, 2020). Given the relevance of internal bodily signals to subjective emotional states, a positive relationship between interoceptive sensibility and emotion regulation was anticipated. Alexithymia could be characterized by a fundamental deficit of interoception, which restricts awareness and self-regulation of emotions but also limits awareness of internal cues of overconsumption, with either or both mechanisms promoting risky or problematic alcohol use. Predictions based on these hypothesized mechanisms were assessed via validated self-report measures of alcohol use, alexithymia, emotion regulation, and interoceptive sensibility.

Based on the two proposed mechanisms of the positive association of alexithymia with alcohol use, indices of either or both deficient emotion regulation and a general deficit of interoception (encompassing non-affective interoception) in alexithymia were expected to mediate the association of alexithymia with alcohol use in young adults. Another trait, reward sensitivity, was presumed to be independently linked to substance use through positive mood enhancement (Lyvers et al., 2018a). The construct of reward sensitivity is typically measured by self-report instruments such as the Sensitivity to Reward subscale of the Sensitivity to Punishment and Sensitivity to Reward Questionnaire (SPSRQ; Torrubia et al., 2001), which was used in the present study. These hypotheses were tested via planned regression and mediation modelling.

## Method

### Participants

The present sample of young adult alcohol users was a subset of a larger sample recruited for a project investigating relationships of alexithymia with addictive behaviors. Approval for the project was granted by the Bond University Human Research Ethics Committee (project no. JT00322) prior to participant recruitment. Participants were recruited through Qualtrics Panels, an online survey hosting tool, and were provided a redeemable points-based incentive (worth approximately $15) by the survey company. Quotas were requested for gender (1:1 male:female) and Australian state of residence proportionate to the population contribution of each state. Inclusion criteria required that participants be aged between 18 and 30 years. Participants were excluded if they were currently taking medication for a neurological or psychological disorder, or had suffered a traumatic brain injury; the reason for this was to reduce extraneous sources of variability in responses.

Data were collected from 572 initial participants; after the survey hosting company removed cases with perseverative responses, missing data, or that did not meet criteria for inclusion, the sample consisted of 541 participants. This was further reduced to 350 participants who reported current alcohol use. Removal of 13 multivariate outliers identified by Mahalanobis distance (*p* < .001) then yielded a final sample of 337 participants aged 18 to 30 years (*M* = 24.77, *SD* = 3.56), of whom 225 (67%) identified as female and 112 (33%) identified as male. There were 224 (66%) non-students and 113 (34%) students in the sample. Highest education level achieved was less than high school for 17 (5%) participants, high school for 119 (35%) participants, undergraduate or trade school degree for 156 (46%) participants, and postgraduate degree for 45 (13%) participants.

### Materials

The following questionnaires were completed by all participants in the final sample.

#### Demographics questionnaire

This consisted of a series of questions requesting information on age, sex, student status, highest education level completed, and (for screening purposes) current use of medication for a psychological or neurological disorder and history of traumatic brain injury.

#### Toronto Alexithymia Scale (TAS-20; Bagby et al., 1994a, b)

The TAS-20 is a 20-item index of alexithymia, with three subscales: difficulty describing feelings (DDF; e.g., “It is difficult for me to find the right words for my feelings”), difficulty identifying feelings (DIF; e.g., “I am often confused about what emotion I am feeling”), and externally oriented thinking (EOT; e.g., “I prefer to just let things happen rather than to understand why they turned out that way”). Respondents indicate the extent to which they agree or disagree with each statement on a five-point Likert scale ranging from 1 (strongly disagree) to 5 (strongly agree). Five items are reverse scored; summing the item responses then yields subscale or total scale scores. Total scores can range from 20 to 100; higher scores indicate higher levels of alexithymia. Scores below 52 are suggested by the scale authors to indicate low or no alexithymia, whereas scores of 52–60 indicate borderline alexithymia, and scores of 61 or higher indicate definite or high alexithymia (Bagby et al., 1994b). Total scores were used in the present study as recommended by the authors of the TAS-20 (see Sekely et al., 2018). The total TAS-20 displayed good internal consistency in the present sample, α = .80.

#### Sensitivity to Punishment and Sensitivity to Reward Questionnaire (SPSRQ; Torrubia et al., 2001)

The SPSRQ is a 48-item questionnaire with two scales: sensitivity to reward (SR) and sensitivity to punishment (SP). The scales assess the influences of the behavioural approach system (BAS; appetitive motivation) and the behavioural inhibition system (BIS; avoidance motivation) respectively, based on Gray’s (1987) influential theory of fundamental brain motivational systems. There are 24 items on the SR scale (even numbered items; e.g., “Do you often do things to be praised?”) and on the SP scale (odd numbered items; e.g., “Are you often afraid of new or unexpected situations?”). Participants respond by ticking either Yes (1) or No (0). Affirmative responses are summed to obtain total scores on SR and SP. Higher scores reflect stronger influences of the corresponding brain motivational systems on everyday behavior. In the present sample, SR and SP showed good internal consistency, with α = .86 for SP and α = .82 for SR.

#### Multidimensional Assessment of Interoceptive Awareness Revised (MAIA-2; Mehling et al., 2018)

The MAIA-2 is a 37-item questionnaire assessing eight dimensions of interoception (more specifically, interoceptive sensibility; Garfinkel et al., 2015) via eight corresponding subscales. The Noticing subscale assesses awareness of bodily sensations whether uncomfortable, neutral, or comfortable (e.g., “I notice when I am uncomfortable in my body”). Not-Distracting assesses the extent to which an individual cannot ignore sensations of discomfort or pain (e.g., “I distract myself from sensations of discomfort,” reverse scored). Not-Worrying assess the tendency to not worry about pain or discomfort (e.g., “I can stay calm and not worry when I have feelings of discomfort or pain”). Attention Regulation assesses the ability to maintain and control attention towards bodily sensations (e.g., “I can return awareness to my body if I am distracted”). Emotional Awareness assesses the emotion-body connection (e.g., “I notice how my body changes when I’m angry”). Self-Regulation assesses the ability to pay attention to body sensations to regulate distress (e.g., “I can use my breath to reduce tension”). Body Listening assesses gaining insight from the body by actively listening to the body (e.g., “Listen to my body to inform me about what to do”). Trusting the Body assesses the body’s signals as reliable (e.g., “I trust my body sensations”). Respondents indicate how often the statements apply to them in daily life, using a six-point Likert scale ranging from 0 (Never) to 5 (Always). Nine items are reverse scored. Subscale scores are calculated by summing responses and dividing by the number of items in each subscale. An overall score can be calculated by summing and averaging all items. Higher scores indicate higher levels of interoceptive awareness. In the present sample, the total MAIA-2 showed high internal consistency, α = .89.

#### Negative Mood Regulation Scale (NMRS; Catanzaro & Mearns, 1990)

The NMRS is a 30-item scale designed to measure confidence in one’s ability to reduce distress via emotional self-regulation. Each item response is anchored on a five-point Likert scale ranging from 1 (strongly disagree) to 5 (strongly agree). After the 13 negatively worded questions are reverse scored, total scores are calculated by summation of item responses. Higher scores indicate stronger beliefs in one’s ability to reduce negative moods via emotional self-regulation. The NMRS reportedly showed discriminant validity from social desirability, impulsivity, and depression (Catanzaro & Mearns, 1990). The NMRS had high internal consistency in the present sample, α = .89.

#### Alcohol Use Disorders Identification Test (AUDIT; Babor et al., 2001)

The AUDIT is a widely used screening tool for risky or harmful alcohol use. Items assess frequency of alcohol consumption (3 items), signs of alcohol dependence (3 items), and alcohol-related problems (4 items). Items 1 to 8 are scored on a five-point Likert scale from 0 to 4, with different anchors depending on the question (e.g., “How often during the last year have you failed to do what was normally expected of you because of drinking?” with options of Never, Less than Monthly, Monthly, Weekly, and Daily or Almost Daily). Items 9 and 10 use a three-point Likert scale with options of 0 (no), 2 (yes, but not during the last year) and 4 (yes, during the last year). Each item score ranges from 0 to 4, thus total scores can range from 0 to 40. Scores of 8 or more indicate hazardous drinking, and scores of 16 or more indicate harmful drinking (Saunders et al., 1993). The AUDIT showed high internal consistency reliability in the present sample (α = .88).

### Procedure

An online link to the questionnaire battery was provided to those who expressed interest in participating. Participants were first presented with an explanatory statement outlining the purpose of the study as an investigation of personality, body awareness, and health habits such as eating and exercise. The statement included assurances of anonymity of participants’ responses and their right to withdraw from the study at any time without penalty, as well as data storage procedures, contact details of the researchers, and a distress hotline (Lifeline) if they experienced distress from their participation. There was a brief disclaimer stating that some of the questions were sensitive, followed by a question asking if the participant agreed to participate in the study. Those who did not click Yes were immediately released from participation. Those who agreed to participate were presented with the demographic questionnaire first, which included questions assessing whether the participant met inclusion criteria; if their responses indicated they did not, they were automatically exited from the survey and thanked for their time. For those who met inclusion criteria, the demographic questionnaire was completed first, followed by the other measures in uniquely randomized orders per participant. Participants had to answer each item per page before they could proceed to the next page. Estimated completion time was 20 to 30 min, after which participants were thanked for their time.

### Statistical Analysis

Hypotheses were tested by hierarchical regression on AUDIT scores followed by bootstrapped mediation modelling. Demographic variables of age, gender, education level, and student status were controlled in all analyses as alexithymia has been reported to vary by age (Mattila et al., 2006), gender (Levant et al., 2009), and education (Lennartsson et al., 2017). Reward sensitivity was also assessed (via SPSRQ) to be included in the regression model for reasons described earlier; in the present sample both SR and SP scales from the SPSRQ showed positive correlations with alexithymia, thus both were entered prior to alexithymia in the regression model and controlled along with the demographic variables in the mediation tests. The strength of correlations was interpreted based on Cohen’s (1988) conventions where *r* = .10 is weak, *r* = .30 is moderate, *r* = .50 is strong.

## Results

IBM’s Statistical Package for Social Sciences (SPSS) version 28 was used for all analyses except the mediation tests. Skewness and kurtosis were well within +/- 1 for all continuous variables, indicating normal or near normal distributions. A conservative alpha level of .01 was chosen to denote statistical significance. Based on the suggested AUDIT cutoff scores, in the current young Australian adult sample 152 (45%) were low risk drinkers, 97 (29%) were hazardous drinkers, and 88 (26%) indicated potentially harmful drinking.

Means, standard deviations, and Pearson correlations among measures are shown in Table 1. As expected the AUDIT index of alcohol use was significantly positively correlated with the TAS-20 index of alexithymia and the SPSRQ-SR index of reward sensitivity, and was negatively correlated with the NMRS index of emotion regulation; however, AUDIT was uncorrelated with the total MAIA-2 index of interoceptive sensibility and the SPSRQ-SP index of punishment sensitivity. TAS-20 alexithymia showed only a weak negative correlation with MAIA-2 interoceptive sensibility that fell short of significance (*p* = .07), but was significantly positively correlated with reward sensitivity and punishment sensitivity, and negatively correlated with emotion regulation. Interoceptive sensibility showed significant correlations with reward sensitivity and emotion regulation, both of which were positive. When MAIA-2 subscale scores were used instead of the total score, AUDIT was not significantly correlated with any of the eight subscales assessing dimensions of interoception; TAS-20 alexithymia showed only a small positive correlation with the Not Distracting subscale (*r* = .16, *p* = .003), a small negative correlation with Attention Regulation (*r* = −.20, *p* < .001) and a moderate negative correlation with Trusting the Body (*r* = −.27, *p* < .001).

**Table 1:** Means (M), Standard Deviations (SD), and Intercorrelations of Variables (N = 337)

Variable	*1*	*2*	*3*	*4*	*5*	*M (SD)*
1. Alcohol Use	-					10.50 (7.57)
2. Alexithymia	.32**	-				59.29 (10.57)
*p*	< .001					
3. Emotion Reg	−.31**	−.49**	-			97.24 (15.12)
*p*	< .001	< .001				
4. Interoception	−.00	−.10	.22**	-		2.67 (0.66)
*p*	.94	.07	< .001			
5. Sens Reward	.38**	.26**	−.10	.24**	-	11.08 (5.09)
*p*	< .001	< .001	.07	< .001		
6. Sens Punish	.09	.47**	−.38**	.01	.41**	14.57 (5.61)
*p*	.12	< .001	< .001	.86	< .001	

*Emotion Reg* Emotion Regulation, *Sens* Sensitivity, *Punish* Punishment

** *p* < .001

### Regression on Alcohol Use Levels (AUDIT)

Hierarchical regression was conducted on AUDIT scores as an index of alcohol use levels, with demographic variables (age, sex, highest completed education level, student/non-student status) as covariates at step 1, the SPSRQ indices of fundamental approach (SR/BAS) and avoidance (SP/BIS) brain systems at step 2, TAS-20 alexithymia at step 3, and both the NMRS index of emotion regulation and the total MAIA-2 index of interoceptive sensibility at step 4. Step 1 was significant, *R*^*2*^ = .12, *F*(4, 330) = 11.29, *p* < .001, accounting for 12% of variance in AUDIT scores; only sex was significant, reflecting higher AUDIT scores in males (*M* = 13.96, *SD* = 8.12) compared to females (*M* = 8.77, *SD* = 6.66), *t*(335) = 6.25, *p* < .001. Step 2 was also significant, *R*^*2*^ = .22, *Fchange*(2, 328) = 20.71, *p* < .001, explaining an additional 10% of variance; the SR index of reward sensitivity was significant, and sex remained significant. Step 3 was again significant, *R*^*2*^ = .28, *Fchange*(1, 327) = 29.62, *p* < .001; alexithymia accounted for an additional 6.5% of variance and was significant, along with sex and reward sensitivity. Step 4 was also significant, *R*^*2*^ = .33, *Fchange*(2, 325) = 11.54, *p* < .001, explaining an additional 4.7% of variance; the NMRS index of emotion regulation was significant but the contribution of MAIA-2 interoceptive sensibility was virtually zero, while reward sensitivity, alexithymia, and sex remained significant. The overall model was significant, *F*(9, 325) = 17.90, *p* < .001, accounting for 33% of variance in alcohol use. In the final model, reward sensitivity was the strongest (and positive) predictor, followed by sex, emotion regulation (a negative predictor), punishment sensitivity (a negative predictor), and alexithymia (a positive predictor) in descending order. Regression statistics including coefficients are shown in Table 2. The substantial decrease in the standardized coefficient for alexithymia after inclusion of emotion regulation in the model (i.e., from .30 to .21) suggested partial mediation.

**Table 2:** Hierarchical regression on levels of alcohol use, controlling for demographic variables (N = 337)

	Step 1			Step 2			Step 3			Step 4		
	*B*	*SE B*	β	*B*	*SE B*	β	*B*	*SE B*	β	*B*	*SE B*	β
Constant	23.09	3.93		15.27	3.91		−.20	4.71		15.81	5.66	
Age	−.01	.13	−.01	.01	.12	.01	.12	.12	.09	.15	.12	.07
Sex	−5.32	.84	−.33**	−4.40	.84	−.27**	−3.93	.81	−.24**	−3.45	.80	−.21**
Education	−1.18	.55	−.12	−.80	.53	−.08	−.41	.51	−.04	−.23	.50	−.02
Student	−.20	.91	−.01	−.72	.86	−.05	.11	.84	.01	−.07	.82	.00
SensRew				.48	.08	.32**	.47	.08	.31**	.54	.08	.36**
SensPun				.00	.08	.00	−.18	.08	−.14	−.28	.08	−.21**
Alexithymia							.22	.04	.30**	.15	.04	.21**
EmoReg										−.13	.03	−.25**
Interocept										−.36	.56	−.03

*B* unstandardized coefficient, *SE B* standard error of *B*, *β* standardized coefficient, *SensRew* Sensitivity to Reward, *SensPun* Sensitivity to Punishment, *EmoReg* Emotion Regulation, *Interocept* Interoception

**p < .001

When the eight MAIA-2 subscales were entered at step 4 instead of the total score, none of the subscales were significant predictors of alcohol use as measured by AUDIT.

### Mediation of Alexithymia to Drinking Levels by Mood Regulation vs. Interoception

**Fig. 1:**
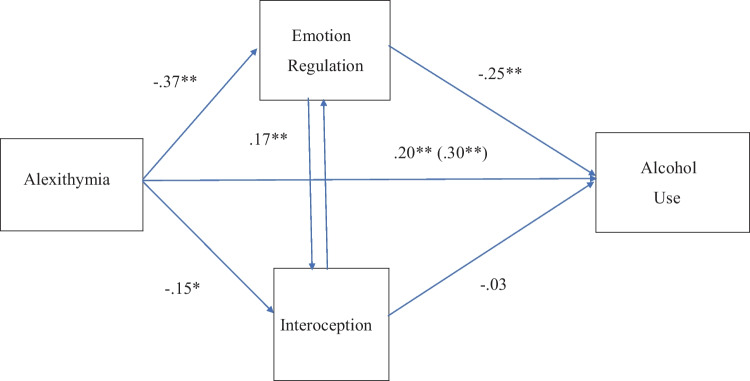
Mediation of the alexithymia – alcohol relationship by emotion regulation but not interoceptive sensibility, controlling for all other variables. The unmediated path is shown in parentheses. *p < .01. **p < .001

The planned mediation test pitting deficient emotion regulation against deficient interoceptive sensibility as hypothesized mediators of the relationship between alexithymia and risky or problematic alcohol use, as measured by AUDIT, was conducted using JASP 0.14.1 with 1000 bias-corrected replications, controlling for age, sex, education level, student status, SR and SP as covariates. Interoceptive sensibility was not a significant mediator as the standardized estimate for the indirect effect was virtually zero; however, emotion regulation was a significant mediator, as the confidence interval for the indirect effect did not include zero (see Table 3). The direct effect was also significant however, indicating partial mediation. Figure 1 depicts the direct and indirect paths. Note that after controlling for all other variables in the path model, a small but significant negative relationship between alexithymia and interoceptive sensibility was indicated, though the latter did not mediate the association of alexithymia with alcohol use levels.

**Table 3:** Mediation of the relationship between alexithymia and levels of alcohol use by deficient emotion regulation but not interoceptive sensibility

**Direct effects**										
									**95% Confidence Interval**	
					**Estimate**	**Std. Error**	**z-value**	**p**	**Lower**	**Upper**
Alexithymia	→	Alcohol Risk			0.21	0.06	3.62	< .001	0.10	0.33

**Indirect effects**										
									**95% Confidence Interval**	
					**Estimate**	**Std. Error**	**z-value**	**p**	**Lower**	**Upper**
Alexithymia	→	EmoRegulation	→	Alcohol Risk	0.09	0.02	3.80	< .001	0.05	0.15
Alexithymia	→	Interoception	→	Alcohol Risk	0.01	0.01	0.63	.53	-.01	0.03

**Total effects**										
									**95% Confidence Interval**	
					**Estimate**	**Std. Error**	**z-value**	**p**	**Lower**	**Upper**
Alexithymia	→	Alcohol Risk			0.30	0.06	5.51	< .001	0.19	0.41

**Total indirect effects**										
									**95% Confidence Interval**	
					**Estimate**	**Std. Error**	**z-value**	**p**	**Lower**	**Upper**
Alexithymia	→	Alcohol Risk			0.10	0.02	3.98	< .001	0.05	0.16

Delta method standard errors, bias-corrected percentile bootstrap confidence intervals, ML estimator

### Mediation of Alexithymia to Emotion Regulation by Interoception

As shown in Fig. 1, interoception and emotion regulation showed the expected significant positive association in the planned model, though the relationship was not strong. A mediation test was conducted such that deficient interoceptive sensibility was examined as a potential mediator of the negative relationship between alexithymia and emotion regulation, based on considerations of theory described earlier. This analysis indicated only a very weak and marginal indirect effect (standardized estimate = −0.03, *p* = .04, CIs = −0.07, −0.01), but a substantial direct effect (standardized estimate = −0.34, *p* < .001, CIs = −0.44, −0.22). The negative association of alexithymia with emotion regulation in the present sample did not appear to be substantially linked to deficient self-reported interoceptive awareness (or sensibility) as measured by the MAIA-2.

## Discussion

The present findings were consistent with the emotion regulation deficit interpretation, but not the general (encompassing non-affective) interoceptive deficit interpretation, of the association between alexithymia and risky or problematic drinking. The AUDIT index of levels of alcohol use showed significant correlations in expected directions with the TAS-20 index of alexithymia, the NMRS index of emotion regulation, and the SPSRQ-SR index of reward sensitivity, but was uncorrelated with the overall MAIA-2 index of interoceptive sensibility (*r* = .00) or its eight subscales. Alexithymia was not significantly correlated with overall interoceptive sensibility, although alexithymia did show small and moderate negative correlations with two of the eight MAIA-2 subscales (Attention Regulation and Trusting the Body, respectively). Alexithymia was however significantly negatively correlated with emotion regulation. Further, the expected positive relationship between interoceptive sensibility and emotion regulation was supported by a small to moderate positive correlation. In a hierarchical regression model, AUDIT scores were predicted by the following variables: sex, such that males reported riskier drinking levels (indicated by higher AUDIT scores); alexithymia and reward sensitivity, as positive predictors of AUDIT scores; and by emotion regulation and punishment sensitivity, as negative predictors of AUDIT scores. Neither the total interoception score nor any of the eight MAIA-2 subscales predicted alcohol use as measured by the AUDIT. A planned mediation test indicated partial mediation of the relationship between alexithymia and alcohol use by deficient emotion regulation but not interoceptive sensibility as indexed by MAIA-2. Further, a second mediation test showed minimal evidence of mediation of the negative relationship between alexithymia and emotion regulation by deficient interoception; instead there was a substantial direct effect. This could suggest that the MAIA-2 index of interoceptive sensibility, despite its comprehensiveness, does not capture aspects of interoceptive sensibility that may be linked to deficient emotion regulation in alexithymia.

The hypothesis proposed by Brewer et al. (2016) that alexithymia represents a fundamental interoceptive deficit, encompassing not only deficient awareness of internal bodily changes associated with emotions but also non-affective internal states including cues of overconsumption (which could also apply to other disorders associated with alexithymia such as binge eating; Aloi et al., 2017), is an appealing idea. It could explain not only the deficits of emotional feelings that define alexithymia, but might also account for the association of alexithymia with risky or problematic alcohol use due to poor awareness of internal cues of overconsumption. However, the findings of the present study did not provide support for this interpretation. Although two of the eight MAIA-2 subscales did show significant negative correlations with the TAS-20 index of alexithymia, none of the others did, and neither did the total score, all of which does not suggest a strong negative relationship between alexithymia and interoceptive sensibility using this measure. More importantly in the present context, there was no evidence that either total MAIA-2 interoception or any of its subscales were related to excessive consumption of alcohol in the present sample of young adult alcohol users. Instead, results indicated a mediating role of deficient emotion regulation in the link between alexithymia and risky or problematic drinking in this sample. The mediation was only partial, however, suggesting that other factors likely contribute to the association of alexithymia with risky or problematic drinking. Possibilities include high rash impulsiveness in alexithymia (Shishido et al., 2013), and/or alcohol expectancies of stronger emotions and assertiveness due to alcohol-induced disinhibition (Thorberg et al., 2016).

The other trait measure that was a significant predictor of alcohol use levels in the final regression model was the SPSRQ with its two scales assessing reward sensitivity (SR scale) and punishment sensitivity (SP scale), the former a positive predictor and the latter a negative predictor. High reward sensitivity has long been considered a risk factor for problematic alcohol or other substance use as noted earlier, hence the former relationship was anticipated; however the negative relationship between punishment sensitivity and alcohol use was unexpected. One possibility is that people with a strong inclination to avoid doing things that might entail punishing consequences tend to refrain from overconsumption of alcohol due to potential negative outcomes such as hangovers, addiction, and adverse impacts on health. A previous study (Lyvers et al., 2013) reported a negative association of SPSRQ-SP with risky or problematic cannabis use and offered a similar interpretation.

### Limitations

Although interoceptive sensibility is likely to play important roles in various psychopathological conditions, a person’s appraisal of their typical level of sensitivity to body signals cannot be assumed to reflect their actual ability to sense such signals; indeed, accuracy and sensibility measures are often dissociable, as discussed earlier. However, also as noted earlier, the common test of interoceptive accuracy, the heartbeat counting task, is compromised by validity issues, thus alternative objective measures are needed. Khalsa et al. (2017) suggested that assessment of sensitivity to experimentally induced internal bodily changes could be used instead of, or in addition to, accuracy tests of resting state interoception; such an approach may be more relevant to the issue of interoceptive sensitivity to emotional changes or alcohol intoxication as well (Lovelock et al., 2021). Although the MAIA appears to be the most comprehensive measure of interoceptive sensibility and was reported by its authors to distinguish in expected ways between experienced and inexperienced mind-body therapy and yoga practitioners (Mehling et al., 2012), the newer version MAIA-2 should be tested against valid objective measures of interoception to evaluate its construct validity. In addition, given the complex multidimensional nature of interoceptive sensibility, Desmedt et al. (2018) advocated using specific subscales to target relevant dimensions, and suggested that the BPQ (Porges, 1993) might be a better index of interoceptive sensibility for neutral and negative internal sensations than the MAIA; such sensations may be particularly relevant in the present context. On the other hand, the MAIA-2 items encompass negative, neutral, and positive sensations, and the total score showed high internal consistency in the present sample. In any case, further work clearly needs to be done on exactly what such purported indices of interoceptive sensibility actually measure, given the divergent findings of studies using such instruments and the generally weak correlations between them.

Self-report indices can be subject to desirability biases and shared method variance in cross-sectional designs, and assume that respondents have accurate knowledge of themselves. People who have limited self-awareness may not be capable of giving accurate responses on self-report measures intended to assess aspects of self-awareness. In particular the use of the TAS-20 can be questioned given that alexithymia is defined by difficulties in perceiving and describing emotional states. However, the TAS-20 has been reported to provide similar assessments of alexithymia to clinician ratings, and some advantages of the TAS-20 over clinician ratings have been reported as well (Ogrodniczuk et al., 2018; Thorberg et al., 2010). Such evidence, combined with its sound psychometric properties, have led to the TAS-20 becoming the most widely used index of alexithymia (Bagby et al., 2020). Nevertheless, Ogrodniczuk et al. suggested that a combination of self-report and clinician ratings should be used to yield the most accurate assessment of alexithymia.

A potentially concerning issue with the present sample was the unusually high mean score on the TAS-20, which indicated borderline alexithymia (Bagby et al., 1994b). Such elevated alexithymia scores have previously been reported in samples recruited online, and were attributed to alexithymic individuals spending more time on the internet given the reported association of alexithymia with higher internet use and internet addiction (Lyvers et al., 2021). Another potential factor to consider however is that the present data were collected during the COVID-19 pandemic when many regions of Australia were subjected to lockdowns, employment-related stress, social distancing, and travel restrictions; corresponding negative impacts on mental health have been documented, especially in young adults (Newby et al., 2020; Rossell et al., 2021). Although much evidence suggests that alexithymia is generally a stable trait with an early developmental onset (Hiirola et al., 2017; Lyvers et al., 2019; Salminen et al., 2006; Tolmunen et al., 2011), a distinction has been made between so-called primary or trait alexithymia and secondary or state alexithymia, with the latter an acute response to depression or stress (Messina et al., 2014); the TAS-20 used in the present study does not distinguish between these types of alexithymia. In any case the unexpectedly high mean TAS-20 score in the current sample may limit the generalizability of the findings. In addition, the current findings were from a nonclinical sample and thus may not apply to clinical AUD samples. Future work could investigate the issues explored in the present study in clinical samples. However, current conceptualizations of addictive behaviors consider them to be distributed continuously in the population, with diagnosed disorders at the extreme end of such distributions (American Psychiatric Association, 2013; Substance Abuse and Mental Health Services Administration, 2016), hence the present findings are likely to be at least somewhat relevant to the general issues investigated.

Finally, the present cross-sectional design could not provide evidence of causal relationships among variables, although in most cases alexithymia is likely to have predated alcohol use given the evidence of its early developmental onset cited earlier. Regardless, the present results do not provide support for the hypothesis that deficient interoceptive sensibility as indexed by MAIA-2 accounts for the association of alexithymia with risky or problematic drinking, because if that were the case then the relationships among the relevant measures would have been consistent with such an interpretation – assuming the measures themselves actually reflect the constructs they are intended to measure (see Desmedt et al., 2022).

## Conclusion

The findings of the present study were consistent with the emotion regulation deficit interpretation of the association of alexithymia with risky or problematic alcohol use in a sample of young adult alcohol users, but did not support a general (encompassing non-affective) interoceptive deficit interpretation - at least with regards to interoceptive sensibility as measured by the MAIA-2. Future work could use validated objective measures of interoceptive accuracy, in addition to self-report indices of interoceptive sensibility, to further evaluate the potential role of interoceptive deficits in the relationship of alexithymia with higher alcohol use. Despite the current negative findings, the intriguing hypothesis of Brewer et al. (2016) that alexithymia is a fundamental deficit of interoception merits further investigation.

## Data Availability

The data for this study are available from the corresponding author on reasonable request.
